# High β-lactam resistance in Gram-negative bacteria associated with kennel cough and cat flu in Egypt

**DOI:** 10.1038/s41598-021-82061-2

**Published:** 2021-02-08

**Authors:** Hazim O. Khalifa, Atef F. Oreiby, Takashi Okanda, Yasuyuki Kato, Tetsuya Matsumoto

**Affiliations:** 1 Department of Infectious Diseases, Graduate School of Medicine, International University of Health and Welfare, Narita, 286-0048 Japan; 2 Department of Pharmacology, Faculty of Veterinary Medicine, Kafrelsheikh University, Kafr El-Sheikh, Egypt; 3 Division of Clinical Research, Medical Mycology Research Center, Chiba University, Chiba, Japan; 4 Department of Animal Medicine (Infectious Diseases), Faculty of Veterinary Medicine, Kafrelsheikh University, Kafr El-Sheikh, Egypt; 5 Department of Microbiology, St. Marianna University School of Medicine, Sugao, Miyamae-ku, Kawasaki, Japan

**Keywords:** Microbiology, Molecular biology

## Abstract

Antimicrobial resistance within pets has gained worldwide attention due to pets close contact with humans. This report examined at the molecular level, the antimicrobial resistance mechanisms associated with kennel cough and cat flu. 1378 pets in total were assessed for signs of respiratory infection, and nasal and conjunctival swabs were collected across 76 diseased animals. Phenotypically, 27% of the isolates were characterized by multidrug resistance and possessed high levels of resistance rates to β-lactams. Phenotypic ESBLs/AmpCs production were identified within 40.5% and 24.3% of the isolates, respectively. Genotypically, ESBL- and AmpC-encoding genes were detected in 33.8% and 10.8% of the isolates, respectively, with *bla*_SHV_ comprising the most identified ESBL, and *bla*_CMY_ and *bla*_ACT_ present as the AmpC with the highest levels. *qnr* genes were identified in 64.9% of the isolates, with *qnrS* being the most prevalent (44.6%). Several antimicrobial resistance determinants were detected for the first time within pets from Africa, including *bla*_CTX-M-37_, *bla*_CTX-M-156_, *bla*_SHV-11_, *bla*_ACT-23_, *bla*_ACT25/31_, *bla*_DHA-1_, and *bla*_CMY-169_. Our results revealed that pets displaying symptoms of respiratory illness are potential sources for pathogenic microbes possessing unique resistance mechanisms which could be disseminated to humans, thus leading to the development of severe untreatable infections in these hosts.

## Introduction

Antimicrobial resistance (AMR) comprises a rapidly emerging growing global threat that exerts enormous economic burdens. Based on recent estimates, AMR’s economic costs varied from £3–11 billion to US $100 trillion^1^. Furthermore, the emergence, widespread occurrence, and spread of antimicrobial resistance among animals, particularly within domestic pets, has raised high levels of global interest. The growing interest among AMR among animals is due to the high possibility of animal-human transmission of highly pathogenic bacteria possessing unique resistance mechanisms such as ESBL, carbapenemases, and *mcr* genes^2–5^. For example, human-specific pathogens have recently been isolated in dogs^4^. Furthermore, pathogenic strains possessing the same extended-spectrum and AmpC beta-lactamases (ESBL/AmpC) have been detected within the same households of humans and dogs^5^. More recently, our research group has identified highly resistant carbapenemase-producing and *mcr-9* coharbouring bacteria associated with pets that had an active respiratory infection that could be transmitted between animals and humans^3^. Within this context, the bacteria associated with respiratory diseases in cats and dogs are of particular importance due to the widespread nature of these contagious diseases, and ease of transmission, in addition to the close proximity of pets and humans.

β-Lactam antibiotics comprise an important group of antibiotics that are widely utilized in human and animal fields, exhibiting strong therapeutic potential. Hence, WHO has identified several β-lactam classes, namely 3rd, 4th, and 5th generation cephalosporins, and carbapenems in addition, as being “critically important” for humans use^6^. Furthermore, several β-lactams are exclusively utilized in the field of veterinary medicine, including ceftiofur and cefquinome, which comprise 3rd and 4th generation cephalosporins^7^. Moreover, in the nation of Egypt, pets are typically treated with antibiotics manufactured for human use, including those forbidden within the veterinary practice, in order to reduce resistance rates. Hence, the heavy use of β-lactam across the veterinary and human practices has acted as a selective pressure facilitating the development of β-lactam resistance. Among the various mechanisms of β-lactam resistance, particular attention had been paid to ESBLs/AmpCs, as these especial enzymes are capable of hydrolyzing penicillins, cephalosporins, and monobactams thus facilitating resistance to multiple antimicrobial agents^2,5,8^. The faulty therapeutic outcomes and high mortality rates associated with ESBL/AmpC producers^8^, have bolstered the scientific interest to elucidate the molecular mechanism of β-lactam resistance, in particular in pets, which act as a potential consignor for human infection^4,5^.

Respiratory diseases in cats and dogs are descriptively known as cat flu and kennel cough. These conditions are complex, with infections caused by various viruses and bacteria, and typically initiated by stress-induced immunosuppression within infected pets^9^. Various Gram-negative bacteria have facilitated these particular challenging diseases and have been reported in several studies to be zoonotic^10^. Such as members of the *Enterobacterales*, alongside *Pseudomonas aeruginosa* that capable of acting as primary or secondary complicating pathogens within kennel cough and cat flu. Other Gram-negative pathogens could possibly also be involved and necessitate transtracheal wash or deep sampling to address their isolation, including *Bordetella bronchiseptica,* which is a commonly occurring bacterium associated with kennel cough and cat flu^11^. At present, a paucity of information is known and available regarding the bacterial resistance mechanisms associated with the respiratory diseases in pets. Hence, the present study was designed to determine the molecular mechanisms of resistance within Gram-negative bacteria isolated from cat flu and kennel cough cases within Egypt and intended to describe the relevant pathogen-specific signs. In our team’s present knowledge, this is the first report investigating the antimicrobial resistance mechanisms associated with respiratory diseases in pets.

## Results

### Clinical features associated with the isolates

Out of the 875 studied cats, 47 specimens (44 nasal swabs and 3 conjunctival swabs) were collected from 44 animals with symptoms of respiratory disease in a sample fraction equal to 5% (95% CI 3.7–6.7%). Thirty-one Gram-negative bacteria were isolated from 24 cats in a percentage comprising 54.5% (95% CI 38.9–69.7%). Various species of Gram-negative bacteria were isolated from the sampled diseased cats, either as singleton or mixed infections. *Enterobacter cloacae* (*n* = 15) occurred the most commonly in isolates from infected cats. followed by *Escherichia coli* (*n* = 10)*,* and *E. fergusonii*, *Klebsiella oxytoca*, *Leclercia adecarboxylate*, *Pantoea septica*, *P. aeruginosa,* and *Raouletella ornithinolytica,* each occurring as a singleton infection from a single animal. Symptoms and their relation to the infecting bacteria are displayed in Table 1.

**Table 1:** Symptoms present in diseased cats.

Infecting bacteria	Infected cases			Associated signs
	Singleton infection	Mixed infection		
		No	Co-infecting bacteria	
*E.* cloacae	10*	–	–	Serous/pale greenish ND NU (5 cases) Sneezing (5 cases) AC of 11 d
	––	2	*E. coli*	Greenish ND NU Conjunctivitis (one case) AC of 9 d in one case and 1.7 y in the other
*E. coli*	6	–	–	Brownish orange/pale Greenish ND NU (1 case) Mild cough (1 case) AC of 6.5 d and 77.5 d in 2 senile animals (13 yrs.)
	–	1	*E. fergusonii*	Serous ND, NU and AC 7 d
	–	1	*P. aeruginosa*	Green ND, NU and AC 7 d
*K. oxytoca*	1	–	–	Serous ND Nu Conjunctivitis AC 9.5 d
*Leclercia adecarboxylate*	1	–	–	
*Pantoea septica*	1	–	–	Green ND NU Conjunctivitis AC 3 d
*Raoultella ornithinolytica*	1	–	–	Green ND NU Conjunctivitis, nasal AC 10 d

*ND* nasal discharge, *NU* nasal ulcer, *AC* average course.

*Two cases were infected by multiple strains of *E. cloacae* with different phenotypic and genotypic resistance patterns (one case infected by 3 strains recovered from different sources and the second case infected with two strains).

Out of the 503 dogs examined in the present study, 37 specimens (32 nasal and 5 conjunctival swabs) were harvested from 32 animals in a sampling fraction of 6.4%. Gram-negative bacteria were isolated from 27 animals (84.4%; 95% CI 67.2%–94.7%). Akin to cats, *E. cloacae* (*n* = 15) was the most commonly occurring Gram-negative bacteria isolated from dogs, followed by *E. coli* (*n* = 12), *K. pneumoniae* (*n* = 4), *Citrobacter braakii,* and *R. ornithinolytica* (*n* = 3 each), *C. freundii* (*n* = 2), and *E. hormaechei, C. farmeri, K. oxytoca,* and *Serratia marcescens* (*n* = 1 each).

Symptoms and their relation to the infecting bacteria are displayed in Table 2. The results of multinomial logistic regression showed that there is a significant association between the older ages and longer course of the disease (*P* < 0.05) (see the “Statistical analysis” section in the supplemental material). Furthermore, cats were almost significantly associated with shorter course than dogs (*P* < 0.08). On the other hand, there was no significant association found between the course of illness and isolate type (*P* > 0.05). There was no significant association either between animal species and specific type of isolates (*P* > 0.05) or between animal age and isolate type (*P* > 0.05). There was no significant association between the type of isolate and associated clinical signs except for *E. cloacae* which was found to be significantly associated with sneezing and ocular discharge (*P* < 0.05).

**Table 2:** Symptoms present in diseased dogs.

Infecting bacteria	Infected cases			Associated signs
	Singleton infection	Mixed infection		
		No	Co-infecting bacteria	
*E.* cloacae	7*	–	–	Serous ND Greenish ND (2 cases) Conjunctivitis (2 cases) Cough (2 cases) Wide range course (3 to 210 d) AC 75.6 d
	–	3	*E. coli*	AC 8.6 d Serous ND NU, Conjunctivitis (1 case)
	–	1	*C. farmeri*	Serous ND Conjunctivitis AC 23 d
	–	1	*K. pneumoniae* *R. ornithinolytica*	
	–	1	*C. freundii*	
*E. coli*	6^¶^	–	–	Serous ND Bloody ND (1 case) Conjunctivitis (2 cases) Cough (1 case) AC 36.1 d
	–	1	*R. ornithinolytica*	Serous ND AC 30 d
*K. pneumoniae*	2	–	–	Greenish ND Severe cough Abnormal lung sounds (1 case) AC 7 days
	–	1	*C. braakii* *E. hormaechei* *R. ornithinolytica*	Serous ND Conjunctivitis Recurrent symptoms in between 2 months
*C. braakii*	1	–	–	Serous ND AC 7 d
	–	1	*C. freundii*	Serous ND Conjunctivitis AC 1 y
*K. oxytoca*	1	–	–	Bloody ND NU AC 30 d
*S. marcescens*	1	–	–	Serous ND Unknown course

*ND* nasal discharge, *NU* nasal ulcer, *AC* average course.

*Two cases each were infected by two *E. cloacae* strains with different phenotypic and genotypic resistance patterns.

^¶^Two cases each were infected by two *E. coli* strains with different phenotypic and genotypic resistance patterns.

### Phenotypic characterization of the isolates

**Figure 1:**
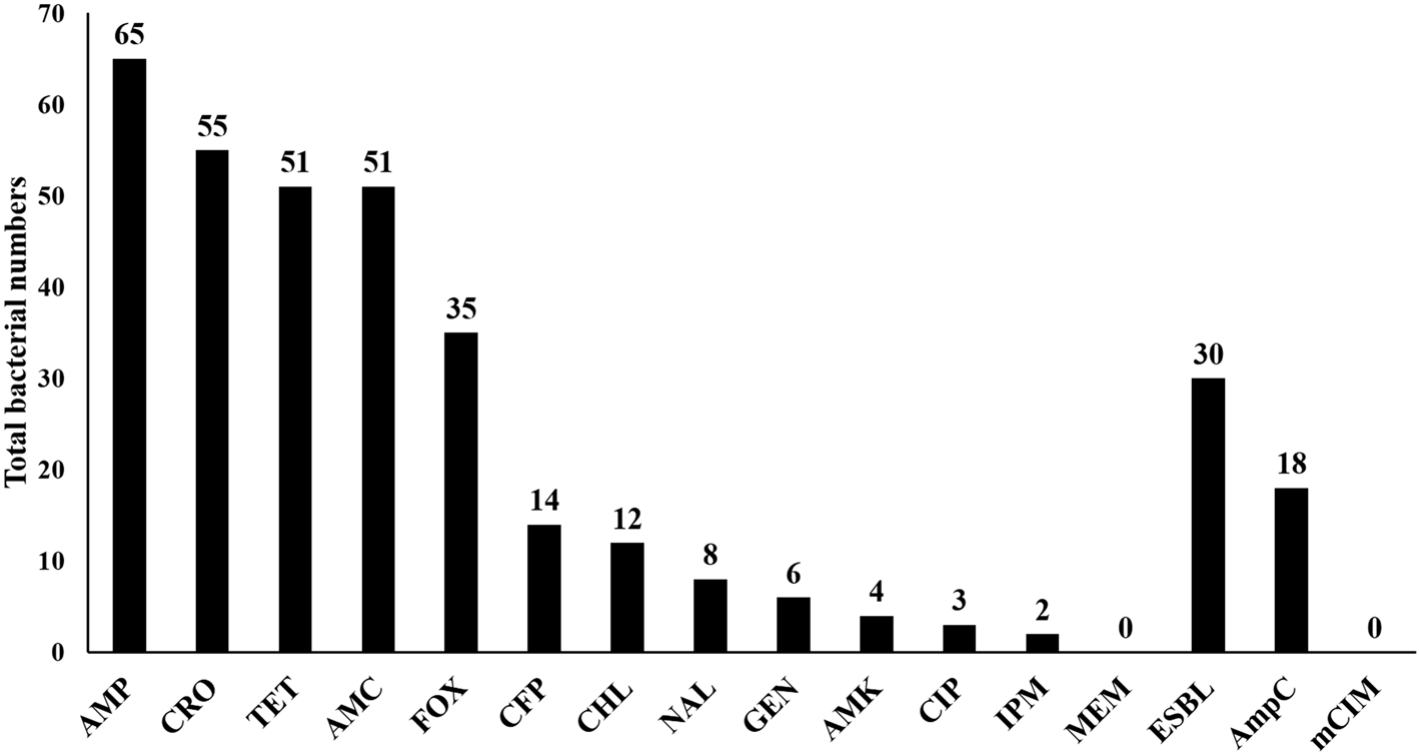
Level of antimicrobial resistance to different antibiotics and phenotypic characters of the isolates. The isolates showed high resistance rates to tetracycline and almost all tested β-lactam antibiotics, except carbapenems with low resistance rates to other tested antibiotics. Phenotypic ESBLs and AmpCs production were detected within 40.5% (95% CI 29.3–52.6%), and 24.3% (95% CI 15.1–25.7%) of the isolates, respectively. On the other hand, all the isolates were negative for phenotypic carbapenemases production according to modified carbapenem inactivation assay (mCIM). Antibiotics: Ampicillin (AMP), ceftriaxone (CRO), tetracycline (TET), amoxicillin–clavulanic acid (AMC), cefoxitin (FOX), cefoperazone (CFP), chloramphenicol (CHL), nalidixic acid (NAL), gentamicin (GEN), amikacin (AMK), ciprofloxacin (CIP), imipenem (IPM), and meropenem (MEM).

A multi-drug resistance profile, based on resistance to at least three antimicrobial classes was observed in 27% of the isolates (*n* = 20) (95% CI 17.4–38.6%). With the exception of carbapenems, the isolates demonstrated high resistance rates to almost all the β-lactam tested (Fig. 1, Supplementary Table S1). In regards, the isolates demonstrated resistance rates by 87.8%, 74.3%, 68.9%, 47.3%, and 19% for ampicillin (AMP), ceftriaxone (CRO), amoxicillin–clavulanic acid (AMC), cefoxitin (FOX), and cefoperazone (CFP), respectively (Fig. 1). Furthermore, the isolates also displayed a high resistance rate to tetracycline (TET) by 68.9%. However, the isolates exhibited low resistance rates to chloramphenicol (CHL, 16.2%), nalidixic acid (NAL, 10.8%), gentamicin (GEN, 8.1%), amikacin (AMK, 5.4%), ciprofloxacin (CIP, 4.1%), imipenem (IPM, 2.7%), and all isolates were susceptible to meropenem (MEM) (Fig. 1).

Of note, phenotypic ESBL production was detected within 40.5% (*n* = 30) (95% CI 29.3–52.6%) of the isolates, and 24.3% (*n* = 18) (95% CI 15.1–25.7%) of the isolates were phenotypically positive to AmpC production (Fig. 1, Supplementary Table S1). However, all the isolates were negative for carbapenemase production via modified carbapenem inactivation (mCIM) assay (Fig. 1, Supplementary Table S1).

### Prevalence of ESBL-encoding genes

**Figure 2:**
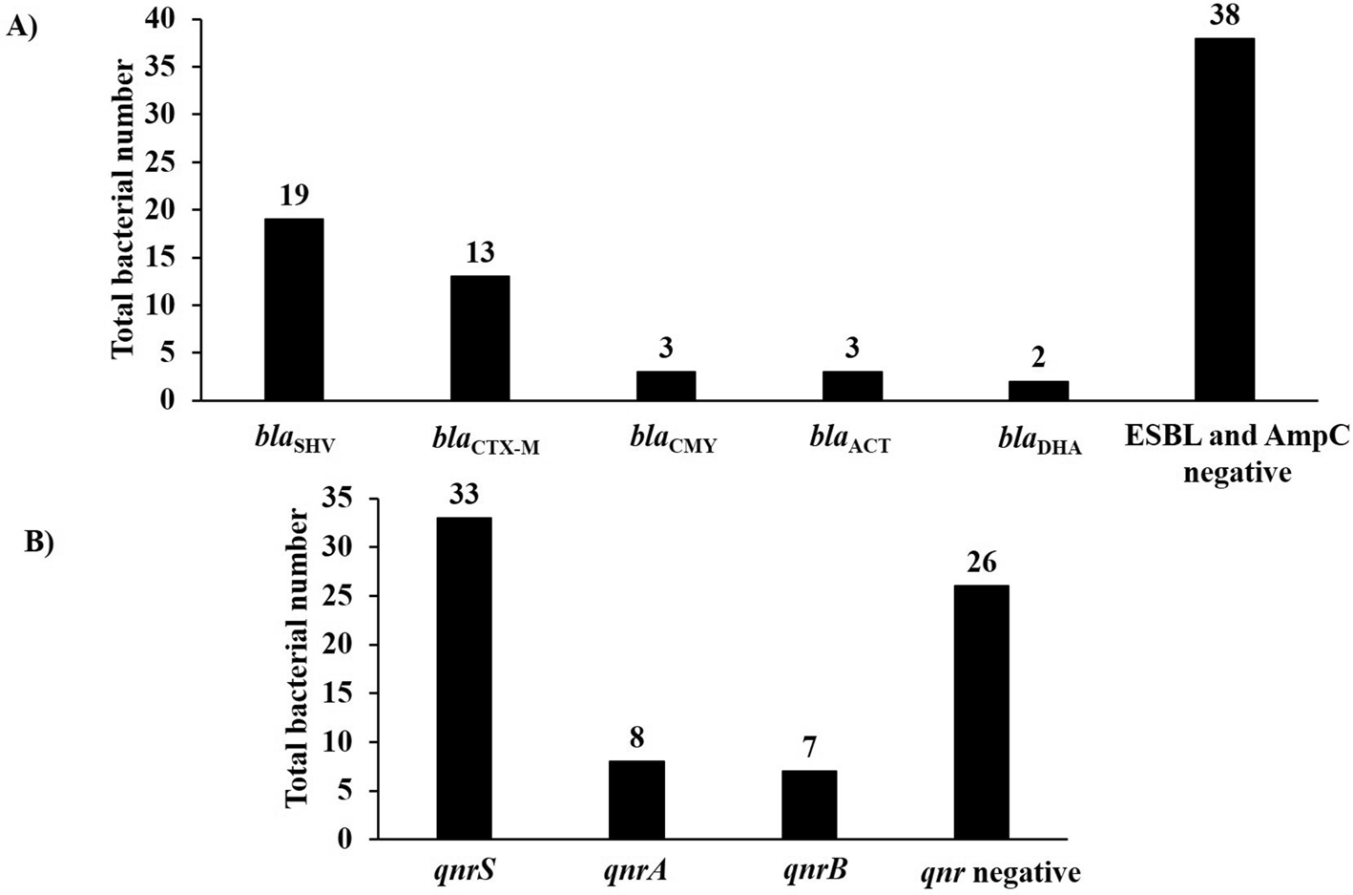
Molecular analysis of ESBL- and AmpC-encoding genes (**A**) and plasmid-mediated quinolone resistance genes (**B**). (**A**): *bla*_SHV_ was the most prevalent ESBL-encoding gene (25.7%; 95% CI 16.2–37.2%) followed by *bla*_CTX-M_ (17.6%; 95% CI 9.7–28.2%). Furthermore, 10.8% (95% CI 4.8–20.2%) of the isolates contained varying AmpC-encoding genes with *bla*_CMY_ and *bla*_ACT_ identified ones within three isolates for each and two isolates were confirmed to possess *bla*_DHA-1_. (**B**) The plasmid-mediated quinolone resistance genes were identified within 64.9% (95% CI 52.9–75.6%) of the isolates, with *qnrS* was the most prevalent (44.6%; 95% CI 33.02–56.6%) followed by *qnrA* and *qnrB* detected within 10.8% and 9.5% of the isolates, respectively.

Varying ESBL-encoding genes were identified via PCR screening and DNA sequencing within 33.8% (25/74) (95% CI 23.2–45.7%) of the isolates (Fig. 2A, Supplementary Table S1). The ESBL with the highest abundance was *bla*_SHV,_ which was identified within 25.7% (95% CI 16.2–37.2%) of the isolates; 18 isolates contained *bla*_SHV-12;_ a single isolate possessed *bla*_SHV-11_. The second most prevalent ESBL-encoding gene was *bla*_CTX-M,_ which was present within 17.6% (95% CI 9.7–28.2%) of the isolates, while five isolates contained *bla*_CTX-M-15_; four isolates contained *bla*_CTX-M-14_; 2 isolates contained *bla*_CTX-M-37_; 2 isolates possessed *bla*_CTX-M-156_. Nine isolates (12.2%; 95% CI 5.7–21.8%) contained *bla*_TEM_, while all isolates were confirmed to be *bla*_TEM-1_ which is not an ESBL-encoding gene. Five isolates registered as phenotypically positive for ESBL production, while genotypic screening confirmed that the isolates were either negative (*n* = 4) or contained *bla*_TEM-1_ (*n* = 1). It follows, the isolates may also possess other unchecked or rare ESBL-encoding genes.

### Prevalence of AmpC-encoding genes

Multiplex PCR followed by single PCR and sequencing confirmed that 10.8% (8/74) (95% CI 4.8–20.2%) of the isolates contained varying AmpC-encoding genes. *bla*_CMY_ and *bla*_ACT_ comprised the most prevalent identified ones within three isolates for each; two isolates contained *bla*_CMY-169_ (G492A); two isolates contained *bla*_ACT25/31_; a single isolate possessed *bla*_CMY-2;_ a single isolate contained *bla*_ACT23_. Two isolates were confirmed to possess *bla*_DHA-1_ (Fig. 2A, Supplementary Table S1). Ten isolates registered as positive for phenotypic detection of AmpC production, while they were negative via PCR screening. These particular isolates may possess other rare or unchecked AmpC-encoding genes.

### Prevalence of other resistance genes

The plasmid-mediated quinolone resistance genes (PMQR) were characterized within 64.9% (48/74) (95% CI 52.9–75.6%) of the isolates (Fig. 2b, Supplementary Table S1). The most prevalent *qnr* gene detected within this study comprises *qnrS,* found in 44.6% (33/74) (95% CI 33.02–56.6%) of the tested isolates. The second most prevalent *qnr* is *qnrA* detected within 10.8% of isolates, followed by *qnrB,* found within 9.5% of isolates. All the isolates were negative for the *qepA* gene and 16S rRNA methylases.

## Discussion

Over the course of the past decade, antimicrobial resistance within the veterinary field has gained worldwide traction due to its important impact upon human health and the growing number of reports of animal-human transmission of antimicrobial resistance^2–5,10^. Within the nation of Egypt, our previous reports regarding AMR within clinical, veterinary, and food settings underscores high levels of resistance, and, in addition, the identification of unique resistance mechanisms within these settings^3,8,12–17^. Furthermore, the unregulated use of antimicrobial agents within the veterinary field, coupled with a lack of data regarding antimicrobial resistance within animals^15^, have resurrected interest driving investigation into their resistance mechanisms, with particular regards to pets. due to the growing concern of resistant bacteria or possible gene transmission between pets and humans.

The results of the present study indicate a widespread prevalence of multidrug-resistant bacteria within pets inflicted with kennel cough and cat flu by 27%, but lower than that previously identified within enteric *E. coli* from domestic pets in Portugal (49.7%)^18^. Furthermore, our team found it surprising that the isolates demonstrated extremely high resistance levels for different β-lactams, in particular AMP (87.7%), CRO (74.3%), AMC (68.9%), and FOX (47.3%,). The ampicillin resistance level is much higher than that previously reported from fecal *E. coli* from Portugal (51.3%)^18^ and Denmark (40%)^19^. Furthermore, β-lactams resistance was also comparable or higher than previously reported within healthy dogs in other European countries including the Netherlands, the United Kingdom, and France^20–22^. The isolates also demonstrated a high resistant rate to tetracycline (69%), which is also much higher than rates previously recorded in Portugal by 45.2%^18^. The widespread prevalence of β-lactam and tetracycline resistance among Gram-negative bacteria associated with respiratory diseases of pets may possibly be attributed to dependence on these drugs as a cheap and readily available remedy for the treatment of pets’ infections^8^. On the other hand, our results demonstrated low levels of resistance to other drugs, in particular for carbapenems, which are strictly utilized only for humans in Egypt^13^, and aminoglycosides and chloramphenicol, which may be due to their limited use, owing to their costs or their complications^8^.

ESBL production is regarded as the major mechanism responsible for β-lactam resistance^2,5,8^. Our results showed that 40.5% of them were phenotypically positive for ESBL production, with 33.8% of the isolates containing at least one ESBL-encoding gene (Supplementary Table S1). Our results are much higher than levels previously identified within *Enterobacterales* from fecal swabs gathered in Spain/France and Switzerland, where 5.3% and 2–2.9% of pets possessed ESBL-encoding genes, respectively^2,23^. Furthermore, our results are comparable to those gathered in China, where phenotypic ESBL production ranged from 24.5% in healthy animals to 54.5% in infected animals^23^. The ESBL genes most widely identified were *bla*_SHV_ and *bla*_CTX-M,_ which is in agreement with previous reports from various countries, such as Spain, France, the Netherlands, the United Kingdom, China, and Tunisia^2,20–22,24,25^. Of note, the majority of the identified *bla*_SHV_ variants is comprised of *bla*_SHV-12,_ which are considered the most widespread among the clinical nosocomial isolates of *Enterobacterales*^26^. Furthermore, *bla*_SHV_ and *bla*_CTX-M_ were also the most widespread within clinical settings and food production in Egypt^8,14^, indicating that they are the major circulating ESBL within Egypt. Although *bla*_CTX-M-15_, *bla*_CTX-M-14_, and *bla*_SHV-12_ were identified from pets in different countries^2,19,21,23^, this is the first report of the identification of *bla*_CTX-M-37_, *bla*_CTX-M-156_, and *bla*_SHV-11_ from pets. Furthermore, this is the first report of *bla*_CTX-M-37_, and *bla*_CTX-M-156_ identification within Egypt and the entirety of Africa.

Of importance, in the present study, we report ESBL-producing *R. ornithinolytica* and *L. adecarboxylate,* and to our present knowledge, this is the first report of their identification stemming from pets in Africa. Both are rare human pathogens and are both associated, but recently are being increasingly reported within clinical cases, which may be associated with severe, life-threatening infections, especially cancer and immunocompromised patients^26,27^. The identification of both pathogens within pets in Egypt poses a considerable public health hazard, with the high possibility of human transmission, facilitating the development of severe, untreatable infections.

The results of the present study demonstrated that 24.3% of the isolates registered as phenotypically positive for AmpC production, with 10.8% carrying AmpC-encoding genes. These results are higher than previously reported levels from pets in the UK, where 16% of the isolates were phenotypically positive, and only 4% harbored *bla*_AmpC_ genes^21^. Furthermore, the prevalence of *bla*_AmpC_ genes identified within this study is much higher than those identified within other European countries, including France/Spain, Denmark, and France, which ranged from 2.1 to 5.4%^2,19,22^. Interestingly, the majority of the identified *bla*_AmpC_ genes from pets were *bla*_CMY-2_, which was detected in pets in France/Spain^2^, Denmark^19^, the Netherland^20^, France^22^, Tunisia^25^, and Italy^28^. On the other hand, *bla*_DHA_ and *bla*_ACT_ are rare *bla*_AmpC_ genes within the field of veterinary medicine, and to the best of our team’s present knowledge, this is the first report of their identification alongside *bla*_CMY-169_ (G492A) from pets in Egypt, the Middle East, and even the entirety of Africa. *bla*_DHA-1_ has been recently identified within pets in Italy^28^, and *bla*_ACT_ gene was identified in Germany^29^.

Quinolones comprise a major group of antibiotics that are commonly used in both human and veterinary medicine and have been identified as one of the most critically important drugs for humans^6^. Due to this fact, in recent years PMQR genes have gained special attention due to their high transmission rates between bacteria via plasmid^8^. Unsurprisingly, our results highlight a high prevalence of *qnr* genes within pets in Egypt by 64.9%, with *qnrS* occurring as the most prevalent (44.6%), supporting our previous reports from clinical settings and food settings in Egypt^8,14^. Our results are much higher than those previously reported within other countries, where *qnr* genes could not be identified in pets from France/Spain and the UK^2,21^. In a recent large-scale study within Europe, *qnr* genes were detected in 20% (32/160) of enrofloxacin non-wild type (resistant) *Enterobacterales*, which represent 3.8% of the total isolates (32/843)^30^. However, *qnr* genes induce low levels of quinolone resistance, but mediate the selection mutations leading to high quinolone resistance^31^. Our results are inconsistent with the previous findings, as 14.3% and 16.3% of *qnr* containing isolates were resistant, and intermediate to nalidixic acid, respectively, and 6.1% and 49.9% of the isolates were resistant and intermediate to ciprofloxacin (data not shown). Our findings confirm our previous studies regarding the lower role of the *qepA* gene and 16S rRNA methylases in the mediation of quinolones and aminoglycosides resistance, respectively^6,8^.

## Conclusion

In summary, the present study reveals wide variation within clinical signs, and a high prevalence of ESBL, AmpC, and PMQR genes across Gram-negative bacteria associated with cat flu and kennel cough within Egypt. Furthermore, unique resistance mechanisms and bacterial species were isolated and identified for the first time within Egypt and the entirety of Africa. Our results are of great clinical concern, as pets could possibly act as a potential reservoir for these antimicrobial resistance determinants that could further disperse them into the human environment. Taking into consideration that the majority of the resistant determinants were previously identified from clinical settings and food in Egypt, the query of animal-human transmission is still needed to be confirmed through future studies.

## Materials and methods

### Animals and sampling

In the present study, a total of 1,378 pet animals, including cats (*n* = 875) and dogs (*n* = 503) hailing from five shelters located in Giza which collect stray pets, as well as those released by their owners in lower and middle Egypt, were selected based upon information regarding outbreaks of respiratory illness. A total of 76 diseased animals (44 cats and 32 dogs) were subjected to precise clinical examination between March and April 2017, and 84 swabs—including 76 nasal swabs and 8 conjunctival swabs—were aseptically collected. All the swabs were transferred to the lab at 4 °C under aseptic conditions for further analysis. All experimental protocols were carried out according to the Kafrelsheikh University Animal Experimentation Regulations, and approved by the Committee on Animal Experiments, Faculty of Veterinary Medicine, Kafrelsheikh University.

### Bacterial isolates

**Figure 3:**
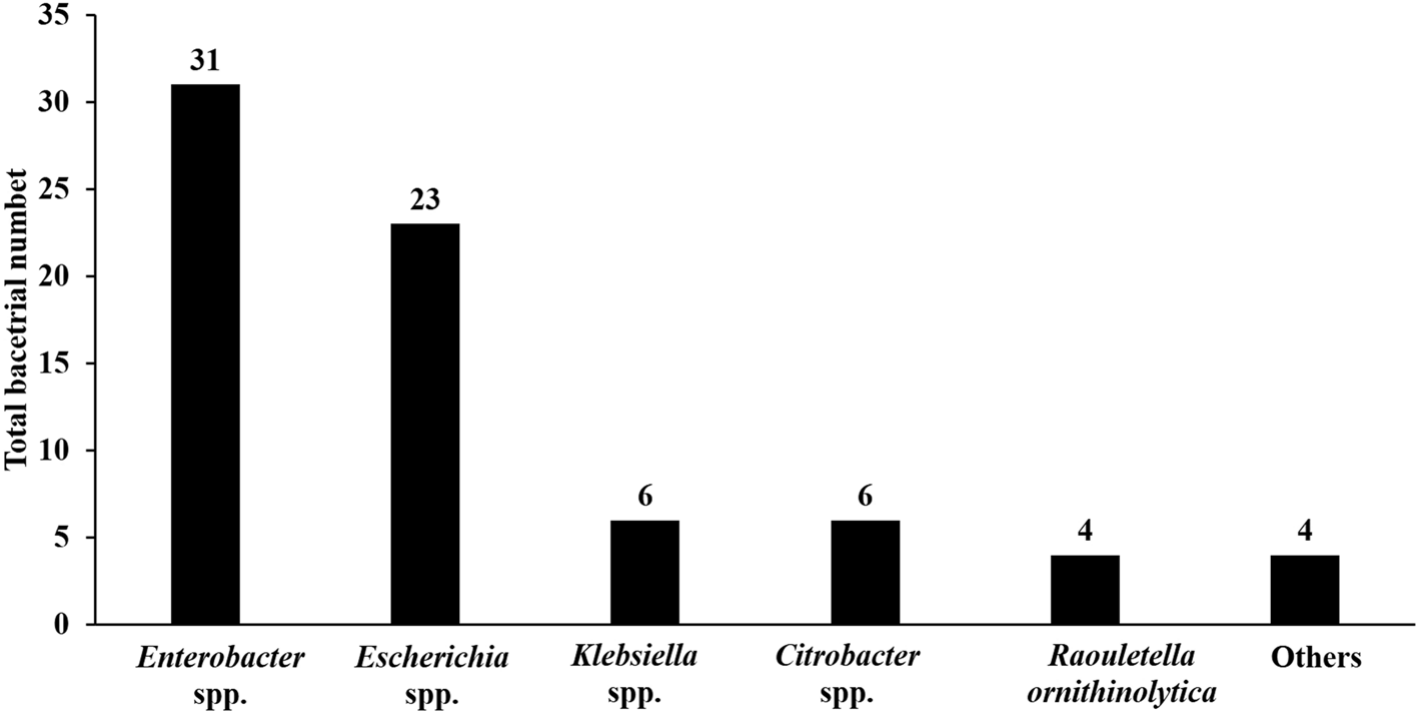
Bacterial isolates identified in this study. *Enterobacter* species include *E. cloacae* (*n* = 30) and *E. hormaechei* (*n* = 1). *Escherichia* species include *E. coli* (*n* = 22) and *E. fergusonii* (*n* = 1). *Klebsiella* species include *K. pneumoniae* (*n* = 4) and *K. oxytoca* (*n* = 2). *Citrobacter* species include *C. braakii* (*n* = 3), *C. freundii* (*n* = 2), and *C. farmeri* (*n* = 1). Others include *S. marcescens* (*n* = 1), *P. septica* (*n* = 1), *L. adecarboxylate* (*n* = 1), and *P. aeruginosa* (*n* = 1).

A total of 74 Gram-negative bacteria were recovered from the nasal cavity (*n* = 65 isolates) and the conjunctiva (*n* = 9 isolates) of 51 pets, including dogs (*n* = 43 isolates) and cats (*n* = 31 isolates), that were tested in this study. All the isolates were identified using MALDI-TOF MS (Matrix Assisted Laser Desorption Ionization-Time of Flight Mass Spectrometry). The isolates were detected to comprise *Enterobacter* spp. (*n* = 31), *Escherichia* spp. (*n* = 23), *Klebsiella* spp. (*n* = 6), *Citrobacter* spp. (*n* = 6), *R. ornithinolytica* (*n* = 4), *Pseudomonas aeruginosa* (*n* = 1), *S. marcescens* (*n* = 1), *L. adecarboxylate* (*n* = 1), and *P. septica* (*n* = 1) (Fig. 3).

### Antimicrobial sensitivity testing

The Kirby–Bauer disc diffusion method was utilized in order to evaluate all of the bacterial isolates for the antimicrobial susceptibility phenotypes to different classes of antibiotics, according to the Clinical and Laboratory Standards Institute (CLSI)^32^. Various antibiotic discs representing the majority of antibiotic families were tested, including AMC (20–10 µg), AMK (30 µg), AMP (10 µg), CHL (30 µg), CRO (30 µg), CFP (75 µg), FOX (30 µg), CIP (5 µg), GEN (10 µg), IPM (10 µg), MEM (10 µg), NAL (30 µg), and TET (30 µg). All of the discs were purchased from Becton, Dickinson and Company Sparks (MD 21,152, USA).

### Phenotypic detection of carbapenemases production

Phenotypic carbapenemase detection was tested via the mCIM method as previously described^33^. Briefly, the bacterial isolates were cultured overnight at 37 °C and a 1-μl inoculation loop was added to 2 ml of tryptic soy broth within a tube. Following 15 s of vortexing, a 10-μg MEM disc was added to the bacterial suspension. The bacterial submission containing the MEM disc was incubated at 37 °C for 4 h. Before the end of the incubation time, the indicator organism (0.5 McFarland standard *E. coli* ATCC 25922) was inoculated at the Mueller Hinton agar plate (MHA). After 4 h incubation of bacterial submission containing the MEM disk, the MEM disk was placed on the inoculated MHA, followed by incubation at 37 °C for 18–24 h. After the incubation period, the result was evaluated by measuring the inhibition zone around the MEM disc.

### Phenotypic detection of ESBL and AmpC production

Phenotypic ESBL and AmpC detection were evaluated utilizing the D68C AmpC and ESBL detection kits (Mast Diagnostics, Mast Group Ltd., Merseyside, U.K.) following the instruction of the producing company and as previously reported^34^. Briefly, the bacterial isolates were cultured overnight at 37 °C on MHA plates, followed by their inoculation at a concentration of 0.5 McFarland standard on MHA plates utilizing a sterile swab. The D68C AmpC and ESBL detection kits contain four different discs, Disc A contains 10 μg of cefpodoxime, disc B 10 μg of cefpodoxime and ESBL inhibitor, disc C 10 μg of cefpodoxime and AmpC inhibitor, and disc D 10 μg of cefpodoxime and both AmpC and ESBL inhibitors. The four discs were placed on the inoculated plates, followed by incubation at 37 °C for 24 h. The final results were evaluated by measuring and contrasting the inhibition zones’ diameters around the four discs. In the phenotypic assays, *E. coli* ATCC 25922 was employed as a negative control and an indicator, while ESBL-, AmpC-, and carbapenemases-producers from our previous reports were employed as positive controls^8,12,13^.

### DNA preparation for PCR experiments

The DNA was prepped by boiling lysates following the method as previously indicated^14^. Briefly, a full 1-μl inoculation loop of bacteria culture incubated overnight on MHA was mixed with 100 µl of sterilized, distilled water, followed by boiling for 8 min. The bacterial suspension was thoroughly shaken then centrifuged, and the supernatant was utilized as a template for PCR experiments.

### Molecular characterization of the isolates

Genetic characterization of the isolates was conducted by PCR experiment and sequencing. The isolates were tested for a wide range of antimicrobial resistance mechanisms via single and/or multiplex PCR as previously described^35–40^ (Supplementary Table S2). The ESBL-phenotypic positive isolates were examined for ESBL-encoding genes (*bla*_CTX-M_, *bla*_SHV_, and *bla*_TEM_) and the AmpC-phenotypic positive isolates were tested for plasmid-mediated AmpC β-lactamase genes (*bla*_ACC_*, bla*_LAT_*, bla*_CMY_*, bla*_BIL_*, bla*_MOX_*, bla*_DHA_*, bla*_MIR_*, bla*_ACT_*,* and *bla*_FOX_)_,_ and all the isolates were checked for plasmid-mediated quinolone resistance genes (*qnrA*, *qnrB*, and *qnrS*), *qepA* gene which is quinolone efflux pump determinant, and 16S rRNA methylases (*rmtA*, *rmtB*, *rmtC*, *rmtD, armA*, and *npmA*).

### Sequencing and data analysis

Target genes were confirmed via single PCR, and PCR fragments were then purified from PCR product or agarose gels using CICA GENEUS PCR & Gel Prep Kit (KANTO CHEMICAL CO., INC., Tokyo, Japan). Following sequencing, the similarity search was performed with the sequenced data using BLAST analysis (http://blast.ncbi.nlm.nih.gov/Blast.cgi).

### Statistical analysis

The association between the course the illness and animal species, isolate type, and the animal age was examined using a multinomial logistic regression model. The association between animal species and the isolate type, age of infected animals and the isolate type, and clinical signs and isolate type were examined using chi-square test. All analyses were carried out using IBM SPSS Statistics for Windows version 21.0. (IBM SPSS Inc, Armonk, NY) and SAS 9.2 (Institute Inc 2008). A *P* value < 0.05 was considered statistically significant. (see the “Statistical analysis” section in the supplemental material).

## Supplementary Information

Supplementary Information
