# Supplementary material for: High β-lactam resistance in Gram-negative bacteria associated with kennel cough and cat flu in Egypt
Source: Sci Rep. 2021 Feb 8;11:3347. doi: 10.1038/s41598-021-82061-2 (PMC7870956; doi:10.1038/s41598-021-82061-2)
Supplement: Supplementary file 1 — Supplementary Information [file 41598_2021_82061_MOESM1_ESM.docx]

**High** **β-lactam resistance in** **Gram-negative bacteria associated with kennel cough and cat flu in Egypt**

**Hazim O. Khalifa^1, 2,3^*, Atef F. Oreiby^4^, Takashi Okanda^5^, Yasuyuki Kato^1^ and Tetsuya Matsumoto^1^**

^1^Department of Infectious Diseases, Graduate School of Medicine, International University of Health and Welfare, Narita, Japan

^2^Department of Pharmacology, Faculty of Veterinary Medicine, Kafrelsheikh University, Kafr El-Sheikh, Egypt

^3^Division of Clinical Research, Medical Mycology Research Center, Chiba University, Chiba, Japan

^4^Department of Animal Medicine (Infectious Diseases), Faculty of Veterinary Medicine, Kafrelsheikh University, Kafr El-Sheikh, Egypt

^5^Department of Microbiology St. Marianna University School of Medicine, Sugao, Miyamae-ku, Kawasaki, Japan

*Address corresponding to Hazim O. Khalifa, Department of Infectious Diseases, Graduate School of Medicine, International University of Health and Welfare, Narita 286-0048, Japan; Phone: +81- 080-3711-8383; FAX: +81-0476-20-7812; E-mail: [hazem.khalifa1@vet.kfs.edu.eg](mailto:hazem.khalifa1@vet.kfs.edu.eg), hazimkhalifa@chiba-u.jp, [omarh_2007@yahoo.com](mailto:omarh_2007@yahoo.com). ORCID: 0000-0001-9861-9693.

**Supplementary Table S1:** **Phenotypic and genotypic characterization of Gram-negative bacteria isolated from pets**

| **No** | **Strain*** | **Species** | **Specimen (Species)** | **Resistance**  **phenotype** | **MDR phenotype** | **Phenotypic ESBL** | **Phenotypic AmpC** | **Phenotypic carbapenemase** | **Identified gene(s)** |
| --- | --- | --- | --- | --- | --- | --- | --- | --- | --- |
| 1 | P15 | *Raouletella ornithinolytica* | Nasal swab (Shirazi  cat) | AMC, AMP, CRO | - | + | - | - | *bla*_CTX-M-15_, *bla*_TEM-1_, *qnrS* |
| 2 | P16 | *E. coli* | Nasal swab (Shirazi  cat) | AMP, CRO | - | - | - | - | *qnrA* |
| 3 | P17 | *E. cloacae* | Nasal swab (Shirazi  cat) | AMP, CRO, FOX | - | - | + | - | *qnrA* |
| 4 | P20-R | *E. coli* | Nasal swab (Shirazi  cat) | AMP, GEN, NAL, TET | + | - | - | - | *qnrA* |
| 5 | P24P | *E. fergusonii* | Nasal swab (Shirazi  cat) | AMC, AMP, FOX, NAL, TET | + | - | - | - | *qnrA* |
| 6 | P24L | *E. coli* | Nasal swab (Shirazi  cat) | AMC, AMP, CHL, GEN, TET | + | - | - | - | *qnrB* |
| 7 | P25 | *E. cloacae* | Nasal swab (Shirazi  cat) | AMP, CRO, FOX | - | + | - | - | *bla*_SHV-12_, *qnrS* |
| 8 | P26N | *E. cloacae* | Nasal swab (Shirazi  cat) | AMC, AMP, CRO, FOX, TET | - | + | + | - | *bla*_SHV-12_, *qnrS* |
| 9 | P27P | *Leclercia adecarboxylate* | Nasal swab (Shirazi  cat) | AMC, AMP, CRO | - | + | - | - | *bla*_SHV-12_, *qnrS* |
| 10 | P28P | *E. cloacae* | Nasal swab (Shirazi  cat) | AMP, FOX, TET | - | - | - | - | *bla*_SHV-12_, *qnrA* |
| 11 | P34 | *E. coli* | Nasal swab (Shirazi  cat) | AMC, AMP, CFP, CRO, TET | - | + | - | - | *bla*_CTX-M-156_, *bla*_TEM-1_, *qnrS* |
| 12 | P38N | *E. coli* | Nasal swab (Baladi  dog) | AMC, AMP, CHL, CRO, TET | + | - | - | - | *qnrB* |
| 13 | P38OL | *E. coli* | Nasal swab (Baladi  dog) | AMP, CFP, CRO, FOX, TET | - | + | - | - | *bla*_CTX-M-15_, *bla*_TEM-1_ |
| 14 | P39O | *C. freundii* | Conjunctival swab (Pitbull dog) | AMC, AMP, CRO, FOX, NAL, TET | + | - | + | - | *bla*_CMY-169_ (G492A), *qnrB* |
| 15 | P39N | *C. braakii* | Nasal swab (Pitbull  dog) | AMP, CRO, CHL, FOX, TET | + | - | + | - | *qnrA, qnrS* |
| 16 | P40 | *E. coli* | Nasal swab (German Shepherd dog) | AMC, AMP, CRO, FOX, TET | - | - | + | - | *bla*_CMY-2_ |
| 17 | P41 | *E. cloacae* | Nasal swab (Baladi  dog) | AMC, AMK, AMP, CRO, TET | + | + | - | - | *bla*_SHV-12_, *qnrS* |
| 18 | P44N | *C. braakii* | Nasal swab (Baladi  dog) | AMC, AMP, CRO, FOX, NAL, TET | + | - | + | - | *qnrA* |
| 19 | P44OL | *E. hormaechei* | Conjunctival swab (Baladi dog) | AMP, CRO, FOX, TET | - | - | + | - | *bla*_ACT-25/31_, *qnrS* |
| 20 | P44OP | *Raoultella ornithinolytica* | Conjunctival swab (Baladi dog) | CRO, TET | - | - | - | - | *qnrS* |
| 21 | P44NL | *K. pneumoniae* | Nasal swab (Baladi  dog) | AMC, AMP, CRO, TET | - | - | - | - | *qnrS* |
| 22 | P45N | *Raoultella ornithinolytica* | Nasal swab (Baladi  dog) | AMC, AMP, CRO, TET | - | + | - | - | *bla*_SHV-12_, *qnrS* |
| 23 | P45R | *K. pneumoniae* | Nasal swab (Baladi  dog) | AMC, AMP, CFP, CHL, CRO, FOX, TET | + | + | - | - | *bla*_SHV-11_, *bla*_CTX-M-15_, *qnrS* |
| 24 | P45W | *E. cloacae* | Nasal swab (Baladi  dog) | AMC, AMP, CFP, CHL, CRO, FOX, TET | + | + | - | - | *bla*_CTX-M-156_, *qnrS* |
| 25 | P46NP | *C. freundii* | Nasal swab (Baladi  dog) | AMC, AMP, CRO, FOX, NAL, TET | + | - | + | - | *bla*_CMY-169_ (G492A), *qnrB, qnrS* |
| 26 | P46OP | *E. cloacae* | Conjunctival swab (Baladi dog) | AMP, CRO, FOX, TET | - | + | - | - | *bla*_SHV-12_, *qnrS* |
| 27 | P48 | *E. cloacae* | Nasal swab (Pitbull  dog) | AMP, CHL, CIP, FOX, GEN, TET | + | + | - | - | *bla*_SHV-12_, *bla*_CTX-M-37_, *qnrS* |
| 28 | P49 | *K. oxytoca* | Nasal swab (Pitbull  dog) | AMP, CHL, CIP, CRO, GEN, TET | + | + | - | - | *bla*_SHV-12_, *qnrS* |
| 29 | P50 | *E. coli* | Nasal swab (German Shepherd dog) | AMC, AMP, CFP, CRO, NAL, TET | + | + | - | - | *bla*_CTX-M-14_, *bla*_TEM-1_, *qnrS* |
| 30 | P50L | *Raoultella ornithinolytica* | Nasal swab (German Shepherd dog) | AMC, CRO, TET | - | + | - | - | *qnrS* |
| 31 | P51O | *E. coli* | Conjunctival swab (German Shepherd dog) | AMC, AMP, CRO, TET | - | - | - | - | *qnrS* |
| 32 | P55 | *K. pneumoniae* | Nasal swab (Baladi dog) | AMC, AMP, CFP, CRO, GEN | - | + | - | - | *bla*_TEM-1B_, *qnrB* |
| 33 | P57R | *E. cloacae* | Nasal swab (Baladi dog) | AMC, AMP, FOX | - | - | + | - | *bla*_ACT-23_ |
| 35 | P58 | *E. coli* | Nasal swab (Baladi dog) | AMC, AMP, CFP, CRO, TET | - | + | - | - | *bla*_SHV-12,_ *bla*_TEM-1_, *qnrS* |
| 36 | P59Y-R | *E. coli* | Nasal swab (Baladi dog) | AMP, CRO, TET | - | + | - | - | *bla*_SHV-12,_ *bla*_TEM-1_, *qnrS* |
| 38 | P59Y-W | *E. cloacae* | Nasal swab (Baladi dog) | AMC, AMP, FOX, TET | - | + | - | - | *qnrS* |
| 39 | P61 | *K. pneumoniae* | Nasal swab (Baladi dog) | AMC, AMP, CRO, FOX, IPM | - | - | + | - | *bla*_DHA-1_ |
| 40 | P62R | *E. coli* | Nasal swab (Baladi dog) | AMP, CIP, NAL, TET | + | - | - | - | *qnrS* |
| 41 | P62W | *E. cloacae* | Nasal swab (Baladi dog) | AMC, AMP, CFP, CRO, FOX, GEN | - | + | - | - | *bla*_CTX-M-15_, *bla*_TEM-1_, *qnrB* |
| 42 | P63P | *C. braakii* | Nasal swab (Baladi dog) | AMC, AMP, CRO, FOX | - | - | + | - | *qnrA* |
| 43 | P66P | *E. cloacae* | Nasal swab (Baladi dog) | AMC, AMK, AMP, CFP, CRO, FOX | - | + | - | - | *bla*_SHV-12_, *bla*_CTX-M-14_, *qnrS* |
| 44 | P66L | *E. cloacae* | Nasal swab (Baladi dog) | AMC, AMP, FOX, TET | - | - | + | - | *bla*_ACT-25/31_, *qnrS* |
| 45 | P68L | *E. coli* | Nasal swab (Baladi dog) | AMC, AMP, CFP, CRO, TET | - | + | - | - | *bla*_CTX-M-15_, *bla*_TEM-1_ |
| 46 | P68P-R | *E. cloacae* | Nasal swab (Baladi dog) | AMC, AMP, CFP, CRO, TET | - | + | - | - | *bla*_SHV-12_ |
| 47 | P69P | *E. cloacae* | Nasal swab (Unknown breed dog) | AMP, CRO | - | + | - | - | *qnrS* |
| 48 | P71 | *E. cloacae* | Nasal swab (unknown breed cat) | AMC, CRO | - | + | - | - | *qnrS* |
| 50 | P72P | *E. cloacae* | Nasal swab (unknown breed cat) | AMC, AMP, AMK, CRO | - | + | - | - | *bla*_SHV-12_, *bla*_CTX-M-14_, *qnrB* |
| 51 | P72L | *E. cloacae* | Nasal swab (Unknown breed cat) | AMC, AMP, CFP, CRO, FOX, TET | - | - | + | - | *bla*_DHA-1_, *qnrS* |
| 51 | P73L | *E. cloacae* | Nasal swab (unknown breed dog) | AMC, AMK, AMP, CRO, TET | + | + | - | - | *bla*_SHV-12_, *bla*_CTX-M-14_ |
| 52 | P73P | *E. cloacae* | Nasal swab (unknown breed dog) | AMC, AMP, CRO, TET | - | + | - | - | *bla*_SHV-12_, *qnrS* |
| 53 | P74 | *E. cloacae* | Nasal swab (unknown breed dog) | AMC, AMK, AMP, CRO, TET | - | + | - | - | *bla*_SHV-12_, *qnrS* |
| 54 | P75 | *E. cloacae* | Nasal swab (unknown breed cat) | AMC, AMP, CFP, CRO, FOX | - | - | + | - | *qnrS* |
| 55 | P76L | *E. cloacae* | Nasal swab (unknown breed cat) | AMC, AMP, CFP, CHL, CRO | - | + | - | - | *bla*_SHV-12_, *bla*_CTX-M-37_, *qnrS* |

**List of abbreviations:** AMC, amoxicillin-clavulanic acid; AMK, amikacin; AMP, ampicillin; AmpC, AmpC beta-lactamases; CFP, cefoperazone, CHL, chloramphenicol; CIP, ciprofloxacin; CRO, ceftriaxone; ESBL, extended-spectrum β-lactamases; FOX, cefoxitin; GEN, gentamicin; IPM, imipenem; MDR, multidrug resistance; NAL, nalidixic acid; TET, tetracycline.

*This table shows the isolates with at least one antimicrobial resistance gene, other 19 isolates without any resistance genes were not listed.

**Supplementary Table S2. Oligonucleotides used in this study**

| Primer name | Sequence (5′-3′) | Target | Reference |
| --- | --- | --- | --- |
| β-lactamases |  |  |  |
| CTXM7  CTXM8 | GCG TGA TAC CAC TTC ACC TC  TGA AGT AAG TGA CCA GAA TC | *bla*_CTX-M-1group_ | 1 |
| CTXM17  CTXM18 | TGA TAC CAC CAC GCC GCT C  TAT TGC ATC AGA AAC CGT GGG | *bla*_CTX-M-2group_ | 1 |
| CTXM19  CTXM20 | CAA TCT GAC GTT GGG CAA TG  ATA ACC GTC GGT GAC AAT T | *bla*_CTX-M-8/25/26group_ | 1 |
| CTXM11  CTXM12 | ATC AAG CCT GCC GAT CTG GTT A  GTA AGC TGA CGC AAC GTC TGC | *bla*_CTX-M-9group_ | 1 |
| SHV_F  SHV_R | AGCCGCTTGAGCAAATTAAAC  ATCCCGCAGATAAATCACCAC | *bla*_SHV-1/variant_ | 2 |
| TEM-F  TEM-R | CATTTCCGTGTCGCCCTTATTC  CGTTCATCCATAGTTGCCTGAC | *bla*_TEM-1/-2/variant_ | 2 |
| MOXMF  MOXMR | GCT GCT CAA GGA GCA CAG GAT  CAC ATT GAC ATA GGT GTG GTG C | *bla*_MOX-1, MOX-2, CMY-1,_  _CMY-8 to CMY-11_ | 3 |
| CITMF  CITMR | TGG CCA GAA CTG ACA GGC AAA  TTT CTC CTG AAC GTG GCT GGC | *bla*_LAT-1 to LAT-4, CMY-2_  _to CMY-7, BIL-1_ | 3 |
| DHAMF  DHAMR | AAC TTT CAC AGG TGT GCT GGG T  CCG TAC GCA TAC TGG CTT TGC | *bla*_DHA_ | 3 |
| ACCMF  ACCMR | AAC AGC CTC AGC AGC CGG TTA  TTC GCC GCA ATC ATC CCT AGC | *bla*_ACC_ | 3 |
| EBCMF  EBCMR | TCG GTA AAG CCG ATG TTG CGG  CTT CCA CTG CGG CTG CCA GTT | *bla*_MIR-1,_ *bla*_ACT-1_ | 3 |
| FOXMF  FOXMR | AAC ATG GGG TAT CAG GGA GAT G  CAA AGC GCG TAA CCG GAT TGG | *bla*_FOX-1 to FOX-5b_ | 3 |
| 16S rRNA methylases |  |  |  |
| armA-F  armA-R | GGTGCGAAAACAGTCGTAGT  TCCTCAAATATCCTCTATGT | *armA*, | 4 |
| npmA-F  npmA-R | CGGGATCCAAGCACTTTCATACTGACG  CGGAATTCCAATTTTGTTCTTATTAGC | *npmA* | 4 |
| rmtA-F  rmtA-R | CTAGCGTCCATCCTTTCCTC  TTTGCTTCCATGCCCTTGCC | *rmtA* | 4 |
| rmtB-F  rmtB-R | GGAATTCCATATGAACATCAACGATGCC  CCGCTCGAGTCCATTCTTTTTTATCAAGT | *rmtB* | 4 |
| rmtC-F  rmtC-R | CGAAGAAGTAACAGCCAAAG  GCTAGAGTCAAGCCAGAAAA | *rmtC* | 4 |
| rmtD-F  rmtD-R | TCATTTTCGTTTCAGCAC  AAACATGAGCGAACTGAAGG | *rmtD* | 4 |
| Plasmid-mediated quinolone resistance |  |  |  |
| qnrA-F | ATTTCTCACGCCAGGATTTG | *qnrA* | 5 |
| qnrA-R | TGCCAGGCACAGATCTTGAC |  |  |
| qnrB-F | CGACCTKAGCGGCACTGAAT | *qnrB* | 5 |
| qnrB-R | GAGCAACGAYGCCTGGTAGYTG |  |  |
| qnrS-F | ACTGCAAGTTCATTGAACAG | *qnrS* | 5 |
| qnrS-R | GATCTAAACCGTCGAGTTCG |  |  |
|  |  |  |  |
| Quinolone efflux pump determinant |  |  |  |
| qepA-F  qepA-R | AACTGCTTGAGCCCGTAGAT  GTCTACGCCATGGACCTCAC | *qepA* | 6 |

**Statistical analysis:**

1. **Results of multinomial logistic regression**

The key is

| Isolate spp | course | age |
| --- | --- | --- |
| 1 = *E.* *cloacae* single infection | 1 less than 7 days | 1 less than 1 year |
| 2 = *E. cloacae* mixed infection | 2 from 8 to 30 days | 2 over 1year to7 years |
| 3 = *E. coli* single infection | 3 from31 to 90 days | 3 older than 7 years |
| 4 = *E. coli* mixed infection | 4 older than 90 days |  |
| 5 = other **G**ram**-**negative bacteria |  |  |

| **Likelihood Ratio Tests** | | | | |
| --- | --- | --- | --- | --- |
| Effect | Model Fitting Criteria | Likelihood Ratio Tests | | |
|  | -2 Log Likelihood of Reduced Model | Chi-Square | df | Sig. |
| Intercept | 81.875 | 10.685 | 4 | 0.030 |
| Isolate | 76.749 | 5.559 | 4 | 0.235 |
| Species | 79.377 | 8.187 | 4 | 0.085 |
| Age | 81.200 | 10.010 | 4 | 0.040 |
| The chi-square statistic is the difference in -2 log-likelihoods between the final model and a reduced model. The reduced model is formed by omitting an effect from the final model. The null hypothesis is that all parameters of that effect are 0. | | | | |

**Parameter estimates**

| course^a^ | | B | Std. Error | Wald | df | Sig. | Exp(B) | 95% Confidence Interval for Exp(B) | |
| --- | --- | --- | --- | --- | --- | --- | --- | --- | --- |
|  |  |  |  |  |  |  |  | Lower Bound | Upper Bound |
| 2 | Intercept | -2.822 | 2.694 | 1.097 | 1 | 0.295 |  |  |  |
|  | Isolate | -0.189 | 0.267 | 0.5 | 1 | 0.48 | 0.828 | 0.491 | 1.397 |
|  | Species | 1.177 | 0.948 | 1.541 | 1 | 0.214 | 3.244 | 0.506 | 20.793 |
|  | Age | 0.794 | 0.852 | 0.869 | 1 | 0.351 | 2.213 | 0.417 | 11.755 |
| 3 | Intercept | -9.527 | 4.906 | 3.771 | 1 | 0.052 |  |  |  |
|  | Isolate | -0.928 | 0.484 | 3.678 | 1 | 0.055 | 0.395 | 0.153 | 1.021 |
|  | Species | 3.281 | 1.568 | 4.378 | 1 | 0.036 | 26.592 | 1.231 | 574.572 |
|  | Age | 2.922 | 1.621 | 3.249 | 1 | 0.071 | 18.581 | 0.775 | 445.564 |
| 4 | Intercept | -9.383 | 4.81 | 3.806 | 1 | 0.051 |  |  |  |
|  | Isolate | -0.24 | 0.438 | 0.3 | 1 | 0.584 | 0.787 | 0.334 | 1.855 |
|  | Species | 1.948 | 1.493 | 1.703 | 1 | 0.192 | 7.012 | 0.376 | 130.712 |
|  | Age | 2.949 | 1.484 | 3.95 | 1 | 0.047 | 19.084 | 1.042 | 349.634 |

^a^The reference category is: 1 for split file $bootstrap_split = 0.

**B. Results of chi-square tests**

**B.1. Species * Isolate**

| **Chi-square tests** | | | |
| --- | --- | --- | --- |
|  | Value | df | Asymptotic Significance (2-sided) |
| Pearson Chi-Square | 4.186^a^ | 4 | 0.381 |
| Likelihood Ratio | 5.343 | 4 | 0.254 |
| Linear-by-Linear Association | 0.714 | 1 | 0.398 |
| N of Valid Cases | 51 |  |  |
| ^a^4 cells (40.0%) have expected count less than 5. The minimum expected count is 1.41. | | | |


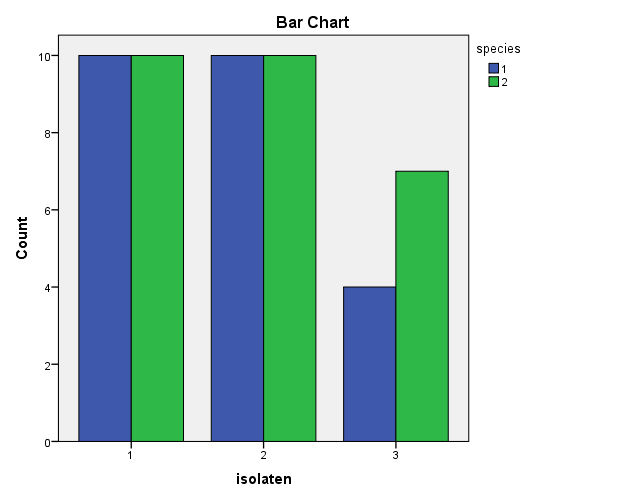


**B.2. Age * Isolate**

| **Chi-square tests** | | | |
| --- | --- | --- | --- |
|  | Value | df | Asymptotic Significance (2-sided) |
| Pearson Chi-Square | 6.163^a^ | 8 | 0.629 |
| Likelihood Ratio | 7.612 | 8 | 0.472 |
| Linear-by-Linear Association | 0.259 | 1 | 0.611 |
| N of Valid Cases | 44 |  |  |
| ^a^11 cells (73.3%) have expected count less than 5. The minimum expected count is .14. | | | |


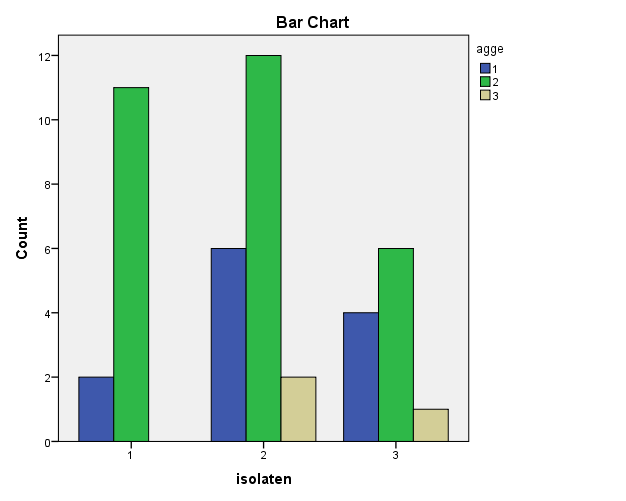


**B.3. Isolate * Nasal discharge**

| **Chi-square tests** | | | |
| --- | --- | --- | --- |
|  | Value | df | Asymptotic Significance (2-sided) |
| Pearson Chi-Square | 2.574^a^ | 4 | 0.631 |
| Likelihood Ratio | 3.416 | 4 | 0.491 |
| Linear-by-Linear Association | 0.000 | 1 | 0.992 |
| N of Valid Cases | 51 |  |  |
| ^a^6 cells (60.0%) have expected count less than 5. The minimum expected count is .41. | | | |


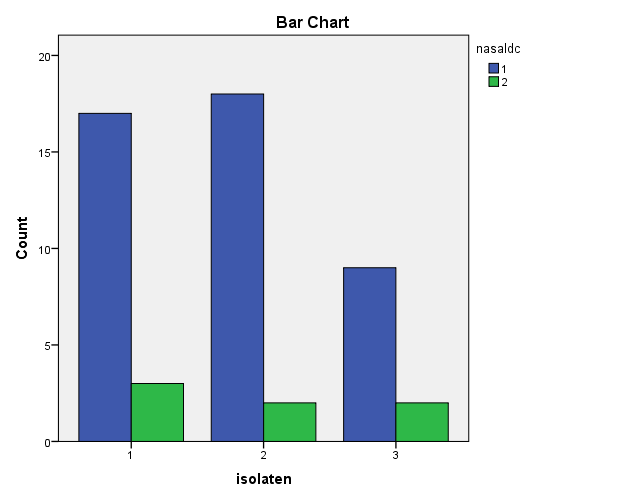


**B.4. Isolate * Nasal ulcer**

| **Chi-square tests** | | | |
| --- | --- | --- | --- |
|  | Value | df | Asymptotic Significance (2-sided) |
| Pearson Chi-Square | 5.541^a^ | 4 | 0.236 |
| Likelihood Ratio | 6.387 | 4 | 0.172 |
| Linear-by-Linear Association | 1.963 | 1 | 0.161 |
| N of Valid Cases | 51 |  |  |
| ^a^5 cells (50.0%) have expected count less than 5. The minimum expected count is 1.06. | | | |


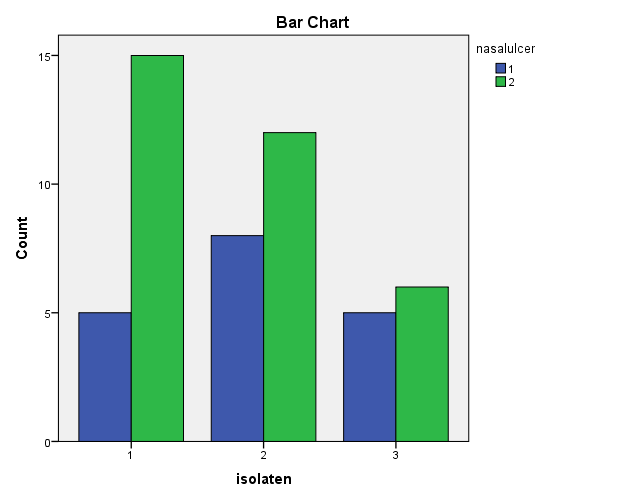


**B.5. Isolate * Cough**

| **Chi-square tests** | | | |
| --- | --- | --- | --- |
|  | Value | df | Asymptotic Significance (2-sided) |
| Pearson Chi-Square | 3.986^a^ | 4 | 0.408 |
| Likelihood Ratio | 5.690 | 4 | 0.224 |
| Linear-by-Linear Association | 1.255 | 1 | 0.263 |
| N of Valid Cases | 51 |  |  |
| ^a^6 cells (60.0%) have expected count less than 5. The minimum expected count is .53. | | | |


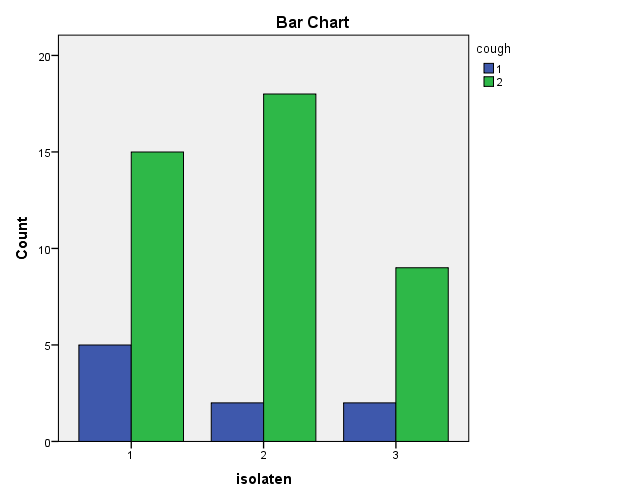


**B.6. Isolate * Sneezing**

| **Chi-square tests** | | | |
| --- | --- | --- | --- |
|  | Value | df | Asymptotic Significance (2-sided) |
| Pearson Chi-Square | 6.728^a^ | 2 | 0.035 |
| Likelihood Ratio | 8.026 | 2 | 0.018 |
| Linear-by-Linear Association | 5.004 | 1 | 0.025 |
| N of Valid Cases | 51 |  |  |
| ^a^3 cells (50.0%) have expected count less than 5. The minimum expected count is .86. | | | |


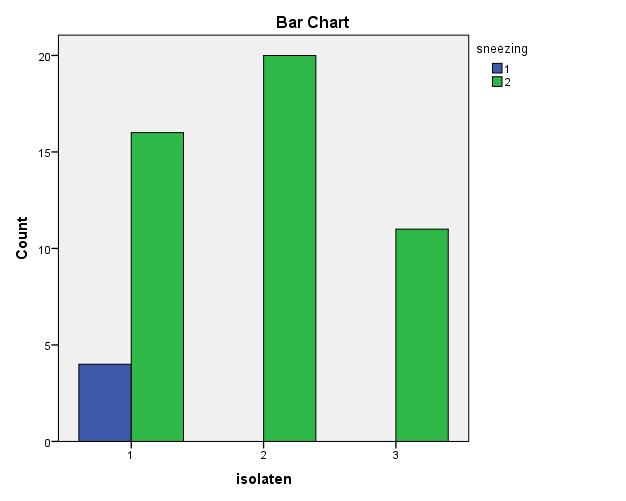


**B.7. Isolate * Ocular discharge**

| **Chi-square tests** | | | |
| --- | --- | --- | --- |
|  | Value | df | Asymptotic Significance (2-sided) |
| Pearson Chi-Square | 6.463^a^ | 2 | 0.040 |
| Likelihood Ratio | 6.661 | 2 | 0.036 |
| Linear-by-Linear Association | 2.091 | 1 | 0.148 |
| N of Valid Cases | 51 |  |  |
| ^a^1 cells (16.7%) have expected count less than 5. The minimum expected count is 4.96. | | | |


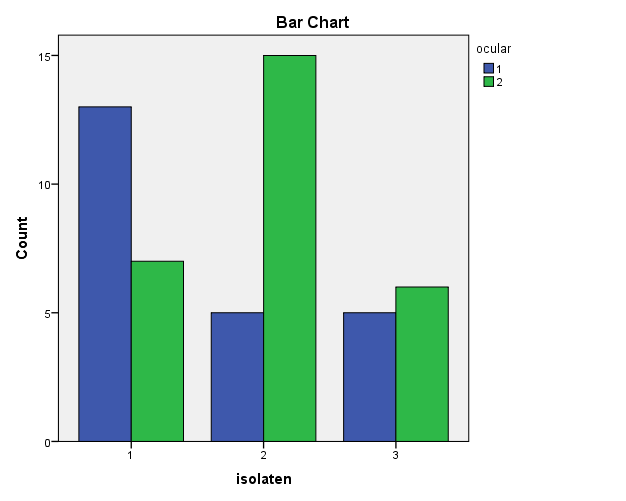


**References**

1. Xu, L., Ensor, V., Gossain, S., Nye K., & Hawkey P. Rapid and simple detection of *bla*_CTX-M_ genes by multiplex PCR assay. J. Med. Microbiol. **54**, 1183-1187 (2005).

2. Caroline D., Anaelle D. C., Dominique D., Christine F., & Guillaume A. Development of a set of multiplex PCR assays for the detection of genes encoding important β-lactamases in *Enterobacteriaceae*. J. Antimicrob. Chemother. **65**, 490-495 (2010).

3. Perez-Perez, F. J. & Hanson N. D. Detection of plasmid-mediated AmpC beta-lactamase genes in clinical isolates by using multiplex PCR. J. Clin. Microbiol. **40**, 2153-2162 (2002).

4. Wangkheimayum et al. Occurrence of acquired 16S rRNA methyltransferase-mediated aminoglycoside resistance in clinical isolates of *Enterobacteriaceae* within a tertiary referral hospital of northeast India. Antimicrob. Agents Chemother. **61**, e01037-16 (2017).

5. Jacoby, G. A., Gacharna, N., Black, T. A., Miller G. H., & Hooper D. C. Temporal appearance of plasmid-mediated quinolone resistance genes. Antimicrob. Agents Chemother. **53**, 1665-1666 (2009).

6. Kim et al. Prevalence of plasmid-mediated quinolone resistance determinants over a 9-year period. Antimicrob. Agents Chemother. **53**, 639-645 (2009).
